# Evaluating Awareness and Practices Pertaining to Radioactive Waste Management among Scrap Dealers in Delhi, India

**DOI:** 10.1371/journal.pone.0091579

**Published:** 2014-03-12

**Authors:** Nayani Makkar, Tany Chandra, Prachi Agrawal, Harshit Bansal, Simranjeet Singh, Tanu Anand, Mannan Kumar Gupta, Rajesh Kumar

**Affiliations:** Maulana Azad Medical College, New Delhi, India; Belgian Nuclear Research Centre SCK/CEN, Belgium

## Abstract

**Objectives:**

With nuclear technology rapidly taking the spotlight in the last 50 years, radiation accidents seem to be a harsh reality of the modern world. The Mayapuri Radiation accident of 2010 was the worst radiation accident India has yet dealt with. Two years thereafter, we designed a study to assess the awareness and practices regarding radioactive waste among scrap dealers aiming to assess deficiencies in radiation disaster preparedness.

**Methodology:**

A community based cross-sectional study. The study population consisted of 209 volunteers (from 108 scrap dealerships) including 108 shop-owners and 101 workers segregated as Group A consisting of 54 dealerships in Mayapuri and Group B of 54 dealerships from the rest of the city. Subjects were then interviewed using a semi-structured questionnaire.

**Results:**

Awareness about radioactive waste varied significantly with level of education (p = 0.024), Kuppuswamy's socio-economic scale (p = 0.005), age of the scrap dealer (p = 0.049) and his work experience (p = 0.045). The larger dealerships in Mayapuri were more aware about radioactive waste (p = 0.0004), the accident in 2010 (p = 0.0002), the symbol for radiation hazard (p = 0.016), as well as the emergency guidelines and the agencies to contact in the event of a radiation accident.

**Conclusions:**

Our findings seem to signify that while governmental and non-governmental agencies were successful in implementing prompt disaster response and awareness programs, the community continues to be inadequately prepared. These go on to suggest that though concerted awareness and training programs do benefit the affected community, economic and social development is the key to disaster prevention and mitigation.

## Introduction

With the emergence of nuclear technologies, radioactivity has become a double-edged sword both of ubiquitous utility and of disparaging detriment to the human race. Over the years much time and energy, of all the worlds' scientists, bureaucrats and medical personnel, has been devoted to ensuring proper management of all possible sources of radioactivity- including appropriate synthesis, transport, use and disposal, with multiple checks and balances to minimize the probability of accidental failure. Once ever so often, though, failure or breech of one or more checkpoints brings the harsh reality - that the accidental or deliberate release of ionizing radiation into the environment is perhaps the biggest modern day threat to humanity [1], [2], [3].

A comprehensive look at radiation accidents, occurring the world over, suggests that such incidents are neither as few nor as far between as the supposed stringency of the security measures in force would seem to suggest. Accidental radiation exposure has occurred in the past in two major forms a) at the site of radioactive source utilization (industrial, research or therapeutic radiation sources which cause accidental overexposure of handlers and/or patients) b) at the site of accidental loss or misplacement of an active source of radioactivity. The second situation, rather implausible from a regulation point of view, occurring due to a lost or ‘orphan’ source of radioactivity, commonly by way of an unsuspecting layman retaining the source with himself in a ‘source-in-pocket’ fashion has in fact led to death and severe injury to members of the general public and caused widespread environmental contamination [1].

### The Mayapuri Radiation Accident, 2010

**Figure 1:**
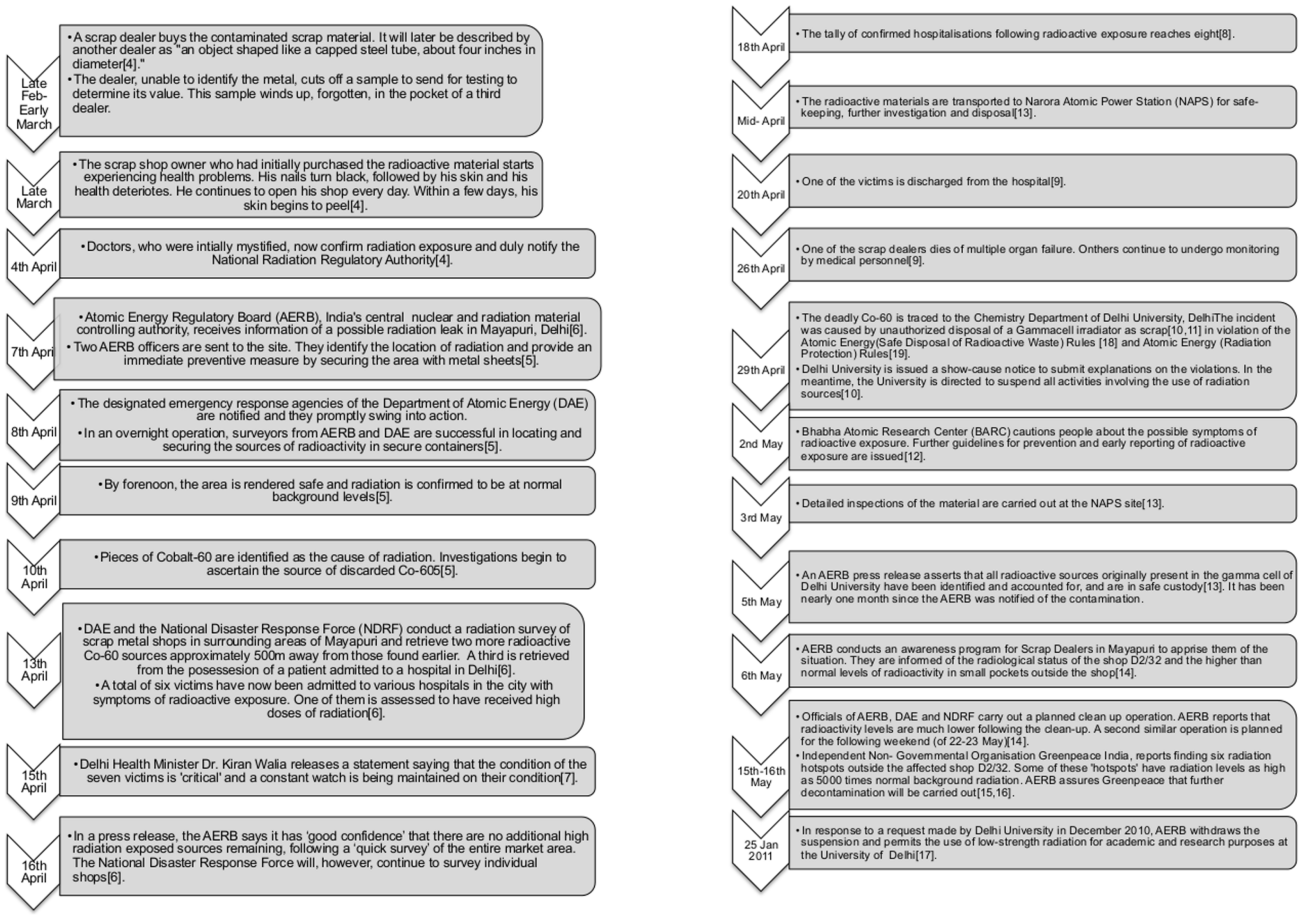
Chronology of events in the Mayapuri Radiation Accident, 2010.

In April 2010, the locality of Mayapuri in New Delhi, India became embroiled in a serious radiation accident [4]–[22]. An AECL (Atomic Energy of Canada Ltd.) Gammacell 220 research irradiator owned by Delhi University since 1968, but lying unused since 1985, was sold at an auction to a scrap dealer in Mayapuri late in February 2010.The Radiation source arrived at a scrapyard in Mayapuri in March, where workers, unaware of the hazardous nature of the device, dismantled it (Figure 1(a)–1(b)). The Cobalt-60 source was cut into pieces exposing many to high levels of radiation: eight people were hospitalized; one succumbed to the injuries [21], [22].

In the National Capital Territory of Delhi, in addition to the governmental agencies involved in collection and disposal of wastes, there is a large and rather complex network of both formal and informal stakeholders involved in the process. Recyclable materials, from households, industries, military auctions, hospitals and laboratories, are collected by rag pickers and scrap merchants and join the recycling stream via the multitude of scrap dealerships functioning independently in the city [23]. By virtue of being the first point of exposure to all forms of discarded metals, scrap dealers, quite ostensibly, form the highest risk group in India for accidental exposure to orphaned sources of radiation. When the radiation accident occurred in 2010, the requirements for the release and disposal of radioactive waste had been clearly mandated as per the Atomic Energy (Safe Disposal of Radioactive Wastes) Rules [18]. However, no legislation had been formulated to regulate the detection and handling of potentially contaminated waste at metal scrap disposal sites [24].

In the disaster mitigation effort that followed the Mayapuri incident, the Bhabha Atomic Research Centre (BARC) and the Atomic Energy Regulatory Board (AERB) conducted information and training programs among the scrap dealers of the Mayapuri scrap colony to generate awareness. The dealers were educated in details of natural and man-made sources of radiation, informed about the importance of undergoing regular medical examination, and trained in the precautionary measures to be taken to prevent radiation hazards in the future [25], [26]. Furthermore, the AERB sought to ‘improve and intensify public awareness in regard to radioactive sources by way of issuing notices through print media and knowledge sharing through the AERB website' [26].

We designed the present study, two years after the Mayapuri incident, to study the awareness and practices regarding radioactive waste and its management among scrap dealers in this locale and the rest of the city of Delhi and assess the preparedness of this at-risk group to manage and mitigate a similar disaster in the future.

## Materials and Methods

### Ethics Statement

Valid, informed written consent was taken from all respondents. Full, free, voluntary consent was taken from each subject. Each respondent was informed of the aim of the study, study procedure, the benefits to be expected from the research, confidentiality of records, their freedom to participate and/or withdraw from the study at any stage, in a language they could fully comprehend. Ethical clearance was obtained from the Institutional Ethical Committee, Maulana Azad Medical College.

A community based Cross-Sectional Study, conducted between two groups of scrap dealerships, with the study group consisting of 54 dealerships in the Mayapuri scrap colony (in close proximity to the proverbial ‘eye-of-the storm’) and the control group consisting of 54 dealerships from all over the city of Delhi (excluding Mayapuri).

Stratified random sampling was used to draw the sample. The government of the National Capital Territory of Delhi has, for ease of administration, divided the region into 9 districts namely Central Delhi, North Delhi, South Delhi, East Delhi, North-East Delhi, South-East Delhi, New Delhi, North-West Delhi, and West Delhi [27]. For the purpose of our study, we selected, one colony of scrap dealerships from each of these districts. Six dealerships were randomly selected from each of these colonies and interviewed for the study.

A pretested semi-structured, in-house questionnaire (included as Questionnaire S1 and Questionnaire S2) was used to interview the owners of scrap dealerships (scrap dealers) and workers at scrap dealerships with respect to their knowledge, attitude and practices pertaining to radioactive waste and its management. The study variables were hence defined to include demographic data (including specific socioeconomic details i.e. age, sex, education level, per-capita family income and Kuppuswamy's socioeconomic score (KS Score)) [28]; as well as details of occupational practices viz. work experience, self-described competency, training in waste identification and/or handling, number of employees at the dealerships, contents and sourcing of scrap material, frequency of general medical check-ups and regularity of agency directed inspections of the dealerships; awareness about radioactive waste (gathered via open-ended questions regarding potential sources, possible health effects, identification of the symbols for radiation hazard and bio-hazard, and self-described possibility for occupational exposure to radioactivity in the future), practices pertaining to radioactive waste management (including availability and deployment of radioactive detection devices, history of detection of and/or exposure to radioactive waste, emergency response agencies for radioactive hazard and their contact details, radiation containment methods/ devices, accessibility of training and emergency guidelines to be followed in the event of a radiation accident) and awareness about the 2010 incident (along with changes in training, safety equipment, radiation monitoring, containment facilities and emergency guidelines, if any, in its aftermath).

Based on data from pilot testing, the required sample size was computed using G*Power 3.1 [29], [30] software. For the average value of degrees of freedom and effect size (as per all demographic variables), the calculated sample size was 207 (for a power of 95% and significance level of 0.05). For ease of divisibility, the study was planned for 216 volunteers. It was subsequently conducted among 209 consenting subjects (from 108 scrap dealerships), comprising of 108 owners of scrap dealerships (54 each from dealerships within and outside Mayapuri) and 101 workers at scrap dealerships (52 from Mayapuri and 49 from dealerships outside Mayapuri).

Subjects were selected from scrap dealerships functioning exclusively in the National Capital Territory of Delhi preferably dealing in scrap that may potentially be contaminated with radioactive material (such as metallic wastes/ motor parts/ hospital / laboratory / industrial / military wastes). All subjects had been working at scrap dealerships in the March of 2010. Dealerships dealing in scrap having little or no risk of being contaminated with radioactive material (such as paper / plastic / wooden / household scrap material) and those operational for a time period of one year or less were excluded from the study, as were workers having a work experience of less than a year.

The statistical analysis of the data thus obtained was performed using a software package, STATA 12 [31]. All demographic variables but two (age and work experience) were discrete. Chi-square tests (or Fisher's exact tests, as appropriate) were applied to each variable functioning as an awareness/ practice parameter to compare it to all candidate variables recording demographic data. Age and work-experience were compared using unpaired two-sample t-tests. Further, all variables were compared between the two study groups i.e. dealerships within Mayapuri and those in other districts. Chi- square tests (or Fisher's exact tests, as appropriate) were applied for the same. ANCOVA was applied with the sum of all awareness parameters as the dependent variable, group (within or outside Mayapuri) as the independent variable, and education, per-capita income and KS score as covariates to statistically control for confounders. Responses gathered from workers were also compared to their demographic characters. Worker responses were further compared between the two study groups (in Mayapuri vs. at other locations).

## Results

### A Comparison of Scrap Dealerships in Mayapuri with other districts across Delhi (findings from the Questionnaire S1 recorded by interviewing owners of scrap dealerships)

All owners of scrap dealerships were apparently healthy males with ages ranging from 16 to 83 years, and duration of work in scrap dealing varying from 2 to 50 years. There was significant variation in the demographic characters of scrap dealership owners within and outside Mayapuri. The level of education was seen to vary significantly between the two sample populations (p<0.0001). While, of dealers in Mayapuri, only one (1.85%) was illiterate and 37.04% were graduates; dealers outside Mayapuri comprised of 14.81% graduates and 12.96% illiterate persons. Dealers in Mayapuri also had higher per-capita income (p = 0.0001). More dealers (9.26%) in Mayapuri belonged to the highest income group (> = INR19575) as compared to 1.85% elsewhere. All dealers belonged to one of three classes on the Kuppuswamy Socio-Economic Scale [28], namely Upper Lower, Lower Middle and Upper Middle. However, dealers in Mayapuri had relatively higher KS Scores (p = 0.0001) [28]. Of dealers in Mayapuri, 50% belonged to the Upper Middle (highest reported) strata as compared to 16.67% dealers elsewhere. Fewer owners of scrap dealerships in Mayapuri participated themselves in the segregation and processing of scrap (p = 0.001). As compared to 20.37% within Mayapuri, fewer (3.70%) dealers outside Mayapuri never handled scrap themselves. Content and sources of scrap received also showed significant variation between the two sample populations. Only dealerships in Mayapuri received scrap from government consignments or scrap comprising machinery. The relatively larger dealerships in Mayapuri were better equipped in terms of Record Maintenance (including records for worker details (p = 0.030) and entry and dispatch of waste (p = 0.031)). A greater proportion of these dealers also actively undertook regular preventive general medical check-ups (p = 0.038).

Awareness about radioactive waste varied significantly between Mayapuri dealers and the others (p = 0.0004). One dealer (1.85%) from Mayapuri as compared to 26% elsewhere had never heard about radioactive waste. Knowledge about the accidental leak in Mayapuri 2010 mimicked the awareness about radioactive waste (p = 0.0002) in that a single dealer (1.85%) in Mayapuri was unaware of the incident in comparison to 27.78% of those outside Mayapuri. Further, more dealers in Mayapuri were aware of the symbol for radioactive hazard (p = 0.016). In Mayapuri, 18.87% dealers were aware of the symbol as compared to 2.5% elsewhere. Dealers within Mayapuri were aware of a wider range of symptoms caused by radioactive exposure. Possible symptoms of radioactive exposure, as reported by them varied significantly between the two sample populations with dealers in Mayapuri reporting hair loss (p = 0.001) and blackening of nails (p = 0.017) as possible effects of radioactive exposure at a higher frequency and a greater predilection of dealers at other locations to report death (p = 0.017). Potential sources of radioactive wastes, as reported by the dealers, also demonstrated a wider and more varied knowledge base in Mayapuri as compared to the other districts.

In the aftermath of the 2010 incident, more dealers in Mayapuri believe they could potentially be exposed to radioactive waste at their workplace in the future (p = 0.007). More dealers (37.74%) in Mayapuri believed they could be exposed to radioactivity at work as compared to 12.5% dealers elsewhere. Six dealers (11.32%), all from Mayapuri, reported the Bhabha Atomic Research Centre (BARC) as the agency/regulatory body managing radioactive waste. A single dealer (1.89%), from Mayapuri, was aware of the standard emergency guidelines, issued by the government, to be followed in the event of accidental radiation leak/ exposure to radioactive material. In the aftermath of the 2010 tragedy, 17 dealerships (32.08%) in Mayapuri reported installation of radiation detection monitors by the government. No dealerships outside Mayapuri had had any form of radiation monitoring exercise. All of the 17 installed monitors have since been removed. Further, only 13.8% of the dealers were regularly using personal protective equipment during handling of scrap and 6.4% had emergency response agency contact details.

(A comparison of the attitude and practices between dealerships within Mayapuri and those outside Mayapuri is presented in Table 1).

**Table 1:** Comparison of Awareness and Practices regarding Radioactive Waste and its management within and outside Mayapuri.

Appropriate Awareness and Practices	Mayapuri (n%)	Outside Mayapuri (n%)	P-value
1. Awareness and Attitude			
a. Heard about radioactive waste	53 (98.15)	40 (74.07)	0.0004
b. Aware of Symbol for radioactivity*	10 (18.87)	1 (2.50)	0.016
c. Aware of controlling agency/ regulatory body for radioactivity/radiation accidents*	6 (11.32)	0	0.0368
d. Aware of the incident at Mayapuri 2010	53 (98.15)	39 (72.2)	0.0002
e. Aware of any other similar incident*	8 (14.81)	9 (16.67)	0.681
f. Believes has an occupational hazard for radioactive exposure*	20 (37.74)	5 (12.50)	0.007
2. Devices and Practices			
a. Radiation Monitor *	17 (32.08)	0	0.0001
b. Available Emergency Guidelines*	1 (1.89)	0	1.0
c. Available Containment Methods*	0	0	-
d. Training following the 2010 accident*	5 (9.43)	8 (21.05)	0.118

*Percentages expressed as a fraction of dealers aware about radioactive waste.

### Variation of Knowledge, Attitude and Practices With Demographic Characters (findings from the Questionnaire S1 recorded by interviewing owners of scrap dealerships)

**Figure 2:**
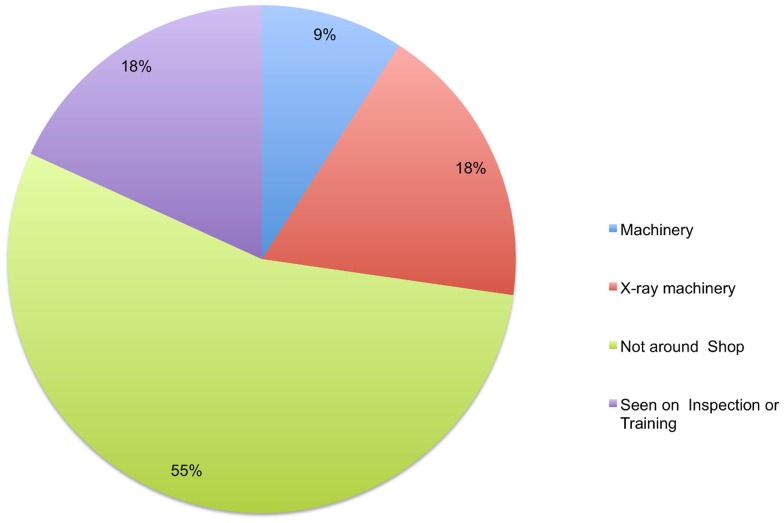
Spatial associations for the symbol for radioactive hazard.

Awareness about radioactive waste was seen to vary significantly with the level of education (p = 0.024), Kuppuswamy's Socio-Economic Score [28] (p = 0.005), age (p = 0.0049) and work experience (p = 0.0452) of the scrap dealer. However, it was NOT seen to vary significantly with the subject's per-capita family income. Akin to that of radioactive waste, awareness about the radiation leak accident in Mayapuri varied with the level of education (p = 0.001), Kuppuswamy's socio economic scale [28] (p = 0.015) and age of the dealer (p = 0.0144). In contrast, however, awareness about this incident did not vary significantly with their work experience. Knowledge regarding the symbol for radioactive hazard varies significantly with Kuppuswamy's Socio-Economic Scale [28] (p = 0.038) and the variation of symbol awareness with per-capita income approached significance (p = 0.052). The spatial associations of the symbol, as reported by aware dealers, have been graphed in Figure 2. Scientific and biomedical research labs, which had been the unwitting sources of the 2010 incident, were cited as a potential source of radioactive waste by more educated dealers at a significantly greater frequency (p = 0.024). Of the illiterate dealers, none were aware whereas 51.85% of graduates/post-graduates were.

Dealers believing they could be exposed to sources of radioactivity at the workplace varied with their per-capita income (p = 0.041). Of dealers belonging to the group with least per-capita income (< = INR 979), 12% (as compared to no dealers in the highest income group (> = INR19575)) held that their situation posed an occupational hazard for accidental future exposure to radioactive material. There was a significant difference in the disaster mitigation measures undertaken in dealerships dealing in ferrous metals as compared to other dealerships. Governmental agencies orchestrated temporary installation of radiation monitors in the shops of 25.49% of scrap dealers dealing in ferrous metals as compared to 7.5% of scrap dealers dealing in other waste (p = 0.025). Further, 22% of ferrous metal dealers as compared 5% others reported training in emergency measures after 2010 (p = 0.023). It is pertinent to note that no scrap dealership was either equipped with or trained to use radiation detection monitors, containment facilities for radioactive waste and/or personal protective equipment.

(The variations in awareness and practices with demographic characters have been summarized in Table 2.).

**Table 2:** Variation of Awareness and Practices regarding Radioactive Waste and its management with the Demographic Characters of the scrap dealers.

Appropriate Knowledge and Attitude	Demographic Character	Trend in Awareness	P-value
1. Heard about radioactive waste	Education Level	Higher among the more educated dealers	0.024
	Per-capita income	-	0.489
	Kuppuswamy Score [28]	Higher in dealers with a higher KS Score [28]	0.005
	Age	Higher in older dealers	0.0049
	Work Experience	Higher among dealers with longer duration of work	0.0452
2. Aware of the incident at Mayapuri 2010	Education Level	Higher among the more educated dealers	0.001
	Per-capita income	-	0.425
	Kuppuswamy Score [28]	Higher in dealers with a higher KS Score [28]	0.015
	Age	Higher in older dealers	0.0144
	Work Experience	-	0.0815
3. Aware of Symbol for radioactivity*	Education Level	-	0.761
	Kuppuswamy Score [28]	Higher in dealers with a higher KS Score [28]	0.038
	Per-capita income	Relatively higher in dealers with higher Per-capita family income	0.052
4. Believes has an occupational hazard for radioactive exposure*	Per-capita income	More dealers with lower Per-Capita Income believe that their job entails an occupational hazard for exposure to radioactivity	0.041

*Percentages expressed as a fraction of dealers aware about radioactive wastes.

### Variation of Knowledge and Practices amongst workers at Scrap Dealerships (findings from the Questionnaire S2 recorded by interviewing workers at scrap dealerships)

Knowledge of the Mayapuri tragedy of 2010 varied significantly amongst the workers with the age (p = 0.0131) (with workers aware of the incident having a higher mean age (31.31 years) as compared to the workers who hadn't heard of it (26.98 years)) and work experience (p = 0.0005) of the worker. Only four workers (all from within Mayapuri) reported initiation of government regulated training programs after the 2010 incident.

## Discussion

As the hub of activity surrounding the events of 2010, Mayapuri served as the tether bringing together seemingly disparate ends of the study. As was expected, awareness about radioactive waste was higher in Mayapuri than in other districts. Furthermore, the possible symptoms of radioactive exposure, as known to dealers within Mayapuri, were largely reminiscent of the 2010 tragedy: more symptoms encountered by the affected population (including hair loss and blackening of nails) were reported. In the aftermath of the incident, more dealers in Mayapuri now believe their job poses an occupational hazard for radioactive exposure.

However, these dealers are also better equipped to handle such an emergency. Only dealers from within Mayapuri were aware of the Bhabha Atomic Research Center as the centralized agency to be reported to in the event of disaster (ironically, most of the dealers knowing about BARC would still choose to report to the police signifying the relative inaccessibility of these specialized agencies to the general populace). Only one of the 54 dealers in Mayapuri was aware of emergency guidelines to be followed in the event of radioactive exposure. In addition, radiation detection monitoring had been conducted within Mayapuri, especially in and around the shops where the incident occurred, in its immediate aftermath. Also, four workers, all from within Mayapuri, had participated in newly devised government-based training programs. Within Mayapuri, a significantly greater number of dealerships that deal in ferrous scrap and receive scrap from government consignments had been subject to monitoring and training activity in the aftermath of the 2010 incident. It becomes especially important that concerted training be undertaken for these dealers, who believe they are at greater risk, to ascertain a maximally efficacious victim response in the event of an accident.

The variation in awareness seems to be multifactorial. It is pertinent to state that dealers in Mayapuri were, on average, better educated, and had a higher per-capita income and better KS score [28]. This does, in part, lay the foundation for their sounder knowledge. Additionally, these findings are suggestive of both witness-based learning and an agency-based response to the 2010 accident. While they do signify some training after the incident, the scope for improvement is great and much work is still required to ensure immunity to such disasters in the future.

Our observations suggest that awareness about radioactive waste and the possible hazards of radioactivity was higher among the more educated dealers as well as those belonging to higher socio-economic groups. There was also a higher level of awareness among both dealers and workers who had been in the profession longer. A mere eleven (10.18%) of the interviewed 108 scrap dealers were aware of the symbol for radioactivity. Again, dealers in higher socio-economic groups and those from within Mayapuri were more aware. Moreover, a majority of the dealers who were aware of the symbol for radioactivity had never encountered it in or around their shop. This may suggest shortcomings in the implementation of radioactive waste disposal guidelines [18] as in terms of properly marking potentially contaminated wastes. Further, only 18% of scrap dealers had seen the symbol during inspection and training. Our findings seem to signify that while governmental and non-governmental agencies were successful in implementing prompt disaster redressal and awareness programs, the community continues to be inadequately informed. These go on to suggest that though concerted awareness and training programs do benefit the affected community, economic and social development is the key to radiation accident prevention and mitigation.

The response program following the 2010 incident left certain fundamental areas of preventive strategy and planning wanting. Not only does the at-risk population require intensive training, but additional facilitation in terms of equipment for personal protection, radiation detection devices, and containment methods. Prevention would also mandate stricter implementation of existing radioactive waste disposal guidelines (including closer monitoring of all radioactive substances and devices being used and conscientious use of the radioactivity symbol) and easier accessibility of centralized radiation control agencies (BARC, AERB).

The study limited itself to scrap dealers and workers in large scrap colonies: individual scrap dealerships were not considered. While the results obtained for dealerships outside Mayapuri should apply to these dealers as well, they do represent a hard to protect segment of the at-risk population. Sampling methods ensured adequate representation of all regions of the city, and of respondents both within and outside Mayapuri. The questionnaire was filled in on the spot by the investigator (as much of the study population had a low literacy level). However, the questions were broad and open-ended to minimize interviewer bias. The possibility of recall bias, however, could not be completely eliminated owing to the retrospective design of the study.

In view of our findings, further study into the adherence to radioactive waste disposal guidelines at source sites and the ability of medical facilities to diagnose and treat acute radiation syndrome would provide insight into the overall preparedness of the system to mitigate future incidents.

## Supporting Information

Questionnaire S1 **Questionnaire used to interview owners of scrap dealerships.**
(PDF)

Questionnaire S2 **Questionnaire used to interview workers at scrap dealerships.**
(PDF)
