# Supplementary material for: Evaluating Awareness and Practices Pertaining to Radioactive Waste Management among Scrap Dealers in Delhi, India
Source: PLoS One. 2014 Mar 12;9(3):e91579. doi: 10.1371/journal.pone.0091579 (PMC3951456; doi:10.1371/journal.pone.0091579)
Supplement: Questionnaire S1 — Questionnaire used to interview owners of scrap dealerships. (PDF) [file pone.0091579.s001.pdf]

Questionnaire S1
Questionnaire for Owners of Scrap Dealerships

I. Identification Data

1. Name:
2. Age:
3. Sex: M / F
4. Residence:
5. Educational Qualification:
6. Occupation: 2
7. Total Family Income:
8. Total number of family members living together:
9. Per-capita income:
10. Type of Family: Nuclear / Joint
11. Socioeconomic status (KS Score):

II. Occupation-related questions:

1. Total Duration of work:
   [If < 1 year, exclude]
   Has any family member of yours been in this business? Y/N
   Has he/she ever suffered from any chronic medical condition? Y/N

2. Do you consider yourself competent to handle scrap? - Y/N
   Have you received any training for handling scrap? - Y/N
   If yes, from where?
   Rank your competency - Competent / Non competent / Don't know

3. Are regular preventive medical check ups being conducted for you? - Y/N
   If yes, how often? every 6 months / 1 year / more than one year

III. Awareness about Radioactive Waste:

1. Have you heard about radioactive waste? Y/N
   If yes, what was the source of information?
   If no, skip to part VI

2. What, in your opinion, are the potential sources of scrap waste contaminated with radioactive material?
   a. Bio medical labs
   b. Hospitals
   c. Some scientific research centers
   d. Mining and processing of Uranium
   e. Naturally Occurring Radioactive Material (NORM) such as coal and oil gas
   f. Industries
   g. Any Other, Specify __________________
   h. All of the Above

2. From which sources of scrap do you believe you could encounter radioactive waste?/
Where do you think scrap material contaminated with radioactive substances could come from? /
What, in your opinion, are the potential sources of scrap waste contaminated with radioactive material?

a. Nausea
b. Burns
c. Hair loss
d. GI syndrome
e. Diarrhea
f. Weakness
g. Cancer
h. Diminished organ function
i. Pneumonitis
j. Cataract
k. Sterility
l. Teratogenic effect
m. Prenatal/ Neonatal death
n. Mental Retardation
o. Genetic mutations
p. All of the Above
q. Ill health/Non-specific
r. Death
s. Photosensitivity
t. Blackening
u. Decreased Cell Counts

3. Are you aware of these symbols: 1* - Y/N, 2* - Y/N
If yes, what do you think it means? 1* -
2* -
Have you seen it in & around your shop, if yes, where? 1* -
2* -

4. Do you think exposure to radioactive waste can have a negative impact on health and/ or cause disease?
If yes, what do you think are effects of radioactive waste on human health and what specific diseases can it cause?

5. Do you think your job entails a risk of accidental exposure to radioactive substances? Y/N
If yes, how?

6. Are you aware of any agency or regulatory body managing radioactive waste? Y/N
If yes, name -

IV. Practices related to Radioactive Waste:

1. Do you have any radiation detection device in your shop? Y/N
If yes, name of device?______________
i) Who handles the machine and what is his qualification?
ii) Does he use personal protective equipment while handling it?
iii) Has he received any special training regarding it?
If no, skip to V

2. When is the material checked for radioactivity?
(a) At arrival of the consignments at the facility
(b) During processing
(c) Final products before dispatch
(d) At all times

3. Have you ever detected radioactive waste? Y/N
If yes, how did you detect it?
What measures did you take?

*Where Symbol 1- Symbol for Radioactive Hazard and
Symbol 2- Symbol for Biohazard

V. In case of nuclear hazard:

1. Reporting:
   a) Whom do you report to?
   b) Are there any phone no available during an emergency?
   c) Mention name & address of agency:
   d) Mode of communication:

2. Containment of the radioactive material till higher authorities take action:
   (a) Is there any special storage facility available? - Y/N
   (b) Does your shop have radiation proof containers available? - Y/N
   (c) Is there any special equipment to handle such waste? - Y/N

3. Are you and/or your worker(s) trained to deal with such emergency? - Y/N

4. Are there any emergency guidelines available? - Y/N

VI. Mayapuri Radiation Hazard Incident:

1. Have you heard about the accidental nuclear leak at Mayapuri in 2010? Y/N
   If yes, what was the source of your information?

2. Have any changes been taken after the incident in your area with respect to:
   (a) Training?
   (b) Safety equipment provided?
   (c) Radiation monitor installed?
   (d) Containment (containers provided / storage facility constructed)?
   (e) Emergency guidelines provided?

3. Have you heard about any other such incident in the past (anywhere)?:
   If yes, please specify:

Observer Check List:

1. Location of shop:
2. Records maintained - Y/N,
   Kinds of records maintained -
   Any specific mention of radioactive waste - Y/N
3. Details of workers in the shop (along with their <u>medical checkup</u>) -
4. Entry of waste (time, date, consignment source) -
5. Maintenance & Calibration of Radioactive Scanning Device -
6. Dispatch of recycled waste (with buyer's name & address) -
7. Inspection of site by some government authority -
8. Presence of Personal Protective Equipment / Working condition -
9. Symbol on Radioactive Waste Bin (if present) -
