# Supplementary material for: Evaluating Awareness and Practices Pertaining to Radioactive Waste Management among Scrap Dealers in Delhi, India
Source: PLoS One. 2014 Mar 12;9(3):e91579. doi: 10.1371/journal.pone.0091579 (PMC3951456; doi:10.1371/journal.pone.0091579)
Supplement: Questionnaire S2 — Questionnaire used to interview workers at scrap dealerships. (PDF) [file pone.0091579.s002.pdf]

Questionnaire S2

Questionnaire for Workers at Scrap Dealerships

I. Identification Data

1. Name:
2. Age:
3. Sex: M / F
4. Residence:
5. Educational Qualification:
6. Occupation: 2
7. Total Family Income:
8. Total number of family members living together:
9. Per-capita income:
10. Type of Family: Nuclear / Joint
11. Socioeconomic status (KS Score):

II. Occupation-related questions:

1. Total Duration of work:
   [If < 1 year, exclude]
   Has any family member of yours been in this business? Y/N
   Has he/she ever suffered from any chronic medical condition? Y/N

2. Do you consider yourself competent to handle scrap? - Y/N
   Have you received any training for handling scrap? - Y/N
   If yes, from where?
   Rank your competency - Competent / Non competent / Don't know

3. Are regular preventive medical check ups being conducted for you? - Y/N
   If yes, how often? every 6 months / 1 year / more than one year

III. Awareness about Radioactive Waste:

1. Have you heard about radioactive waste? Y/N
   If yes, what was the source of information?
   If no, skip to part VI

2. What, in your opinion, are the potential sources of scrap waste contaminated with radioactive material?
   a. Bio medical labs
   b. Hospitals
   c. Some scientific research centers
   d. Mining and processing of Uranium
   e. Naturally Occurring Radioactive Material (NORM) such as coal and oil gas
   f. Industries
   g. Any Other, Specify __________________
   h. All of the Above

3. Are you aware of these symbols: 1* - Y/N, 2* - Y/N
   If yes, what do you think they mean?: 1* -
   2* -
   Have you seen it in & around your shop? Y/N
   If yes, where? 1* -
   2* -
4. Do you think exposure to radioactive waste can have a negative impact on health and/ or cause disease?
   If yes, what do you think are effects of radioactive waste on human health and what specific diseases can it cause?
   a. Nausea
   b. Burns
   c. Hair loss
   d. GI syndrome
   e. Diarrhea
   f. Weakness
   g. Cancer
   h. Diminished organ function
   i. Pneumonitis
   j. Cataract
   k. Sterility
   l. Teratogenic effect
   m. Prenatal/ Neonatal death
   n. Mental Retardation
   o. Genetic mutations
   p. All of the Above
   q. Ill health/Non-specific
   r. Death
   s. Photosensitivity
   t. Blackening
   u. Decreased Cell Counts

5. Do you think your job entails a risk of accidental exposure to radioactive substances? Y/N
   If yes, how?

6. Are you aware of any agency or regulatory body managing radioactive waste? Y/N
   If yes, name –

7. Have you ever come across radioactive waste? Y/N
   i) If yes, how did you detect it?
   ii) What measures did you take?

8. Are you trained to deal with radiation hazard emergency? Y/N

VI. Mayapuri Radiation Hazard Incident:

1. Have you heard about the accidental nuclear leak at Mayapuri in 2010? Y/N
   If yes, what was the source of your information?

2. Have any changes been taken after the incident in your area with respect to:
   (a) Training?
   (b) Safety equipment provided?
   (c) Radiation monitor installed?
   (d) Containment (containers provided / storage facility constructed)?
   (e) Emergency guidelines provided?

3. Have you heard about any other such incident in the past (anywhere)?
   If yes, please specify:

*Where Symbol 1- Symbol for Radioactivity and
Symbol 2- Symbol for Biohazard
